# The Systemic Connection in Dental Erosion: A Cross-Sectional Analysis of the Interrelationship Between General Health Status and Alimentation Patterns

**DOI:** 10.3390/medicina62081575

**Published:** 2026-08-17

**Authors:** Simona Iacob, Mădălina Bălaj, Radu Chisnoiu, Andrea Maria Chisnoiu, Adina Iosa, Mihaela Păstrav, Smaranda Buduru, Andreea Kui

**Affiliations:** 1 Department of Prosthodontics, Faculty of Dental Medicine, “Iuliu Hațieganu” University of Medicine and Pharmacy, 400012 Cluj-Napoca, Romania; iacob.simona@umfcluj.ro (S.I.); madalina.geor.balaj@elearn.umfcluj.ro (M.B.); adina.elen.iosa@elearn.umfcluj.ro (A.I.); dana.buduru@umfcluj.ro (S.B.); gulie.andreea@umfcluj.ro (A.K.); 2 Department of Odontology, Endodontics and Oral Pathology, Faculty of Dental Medicine, “Iuliu Hațieganu” University of Medicine and Pharmacy, 400012 Cluj-Napoca, Romania; marcel.chisnoiu@umfcluj.ro; 3 Department of Orthodontics, Faculty of Dental Medicine, “Iuliu Hațieganu” University of Medicine and Pharmacy, 400012 Cluj-Napoca, Romania; mihaela.manasia@umfcluj.ro

**Keywords:** dental erosion, dietary habits, salivary buffering capacity

## Abstract

*Background and Objectives*: This study aimed to investigate the prevalence and severity of dental erosion using the Basic Erosive Wear Examination (BEWE) index in an adult cohort, exploring its associations with systemic health, gastrointestinal symptoms, and dietary habits. *Materials and Methods*: An observational cross-sectional study was conducted with 170 adult patients at a university clinic in Cluj-Napoca, Romania, between January and June 2026. Participants completed a standardized questionnaire detailing general characteristics, diet, systemic conditions, and symptoms. Dental erosion was clinically recorded via the highest BEWE score per sextant. Chairside tests evaluated unstimulated salivary pH and buffering capacity. Data were analyzed using chi-square tests and ordinal logistic regression. *Results*: Most patients exhibited low BEWE risk. Bivariate analyses revealed significant associations between elevated BEWE risk and advanced age, GERD, eating disorders, and carbonated beverage consumption. However, in the adjusted ordinal logistic regression model, only advanced age, GERD, digestive symptoms (difficulty swallowing, digestive burns), and specific medications (anti-asthma drugs, aspirin) remained independent predictors of increased erosive wear risk. *Conclusions*: Dental erosion is a multifactorial condition strongly shaped by advanced age, GERD, eating disorders, and carbonated beverage intake. A comprehensive risk-based approach incorporating medical histories, symptom screening, and dietary counseling is essential for early clinical identification and prevention.

## 1. Introduction

Dental erosion refers to the permanent loss of enamel and dentin resulting from direct chemical dissolution by non-bacterial acids. In recent years, it has gained recognition as a major contributor to tooth wear observed in both clinical practice and epidemiological research [1,2]. The development of dental erosion is complex and multifactorial, involving interactions between intrinsic systemic conditions, dietary acid exposure, and behavioral factors that collectively modulate the frequency and intensity of acidic challenges as well as salivary protective mechanisms [3,4].

Systemic health status is an important dimension in the development and progression of dental erosion. Disorders characterized by recurrent exposure of the oral cavity to gastric acids, particularly gastroesophageal reflux disease (GERD), generate a persistent intrinsic acidic challenge that may exceed the neutralizing and protective capacity of saliva, thereby promoting enhanced demineralization of enamel. A comprehensive overview of reviews reports a robust association between GERD and erosive tooth wear, highlighting that individuals with GERD exhibit a higher prevalence of dental erosion compared with healthy populations [5]. Comparable mechanisms are observed in patients with eating disorders, where repeated self-induced vomiting results in frequent contact between gastric acid and dental hard tissues. Systematic analyses reveal a notably elevated occurrence of erosive lesions among individuals with bulimia nervosa and anorexia nervosa, with the strongest association reported in bulimic patients, who present substantially greater odds of dental erosion relative to control groups [6,7]. These findings underscore the systemic influences on oral health and support the notion that dental erosion cannot be fully understood without considering underlying general health conditions.

Dietary habits also represent a critical and modifiable contributor to dental erosion. Regular intake of acid-containing foods and beverages, such as citrus-based drinks, carbonated soft drinks, and sports beverages, has repeatedly been linked to a greater occurrence and increased severity of erosive tooth wear [8]. Among individuals affected by gastroesophageal reflux disease, specific dietary practices and lifestyle patterns, including late-evening meals and frequent ingestion of acidic products, further amplify intraoral acid exposure. This repeated acid challenge can reduce salivary pH and compromise buffering efficiency, thereby accelerating enamel demineralization [9]. When external dietary acids act in conjunction with intrinsic gastric acids associated with systemic disorders, a cumulative or synergistic effect on dental hard tissue degradation may occur.

Collectively, these data suggest that the interrelationship between general health status, dietary patterns, and dental erosion is complex and multifactorial. A comprehensive understanding of these interactions is essential for improving early detection, developing targeted preventive strategies, and integrating oral and systemic health management into clinical practice.

The present study aimed to investigate the prevalence and severity of dental erosion, as assessed by the Basic Erosive Wear Examination (BEWE) index, in a cohort of adult patients and to explore its associations with systemic health conditions, gastrointestinal symptomatology, and dietary habits.

## 2. Materials and Methods

An observational cross-sectional study was conducted at the Department of Prosthetic Dentistry, “Iuliu Hațieganu” University of Medicine and Pharmacy, Cluj-Napoca, Romania, over the period January–June 2026. Participants were recruited by consecutive (non-probabilistic) sampling. All patients presenting to the clinic during the study period who met the eligibility criteria and provided informed consent were enrolled.

The study methodology was approved by the Ethics Committee of “Iuliu Hațieganu” University of Medicine and Pharmacy, Cluj-Napoca, Romania (approval no. PNZ36, dated 28 January 2026). All participants provided informed consent prior to enrollment.

The inclusion criteria in the study group were willingness to participate, complete dental arches or at least 3 dental units per sextant, and absence of extensive fixed or removable prosthetic restorations. The exclusion criteria were refusal to participate, total or subtotal edentulism, and extensive fixed or removable prosthetic restorations.

Data were collected through a standardized CAP (Knowledge–Attitudes–Practices) self-completed questionnaire administered prior to the clinical examination, after informed consent was obtained. The questionnaire comprised 12 items with predefined single or multiple-choice response options, documenting participants’ general characteristics, dietary habits, systemic health conditions, and gastrointestinal/respiratory symptoms. Dietary exposure was categorized by frequency of consumption (Never/Occasional/Weekly/Daily). Systemic conditions (asthma, autoimmune diseases, alcoholism, gastroesophageal reflux disease, eating disorders) were coded as dichotomous variables (present/absent), and age was grouped into six categories (<20, 20–30, 31–40, 41–50, 51–60, >60 years).

Dental erosion was evaluated using the Basic Erosive Wear Examination (BEWE) index, as described by Bartlett et al. (2008) [10]. The highest BEWE score per sextant (0 to 3) was recorded, and the cumulative score across all six sextants (ranging from 0 to 18) was classified into risk levels according to standard thresholds: none/no risk (cumulative score ≤2), low risk (cumulative score 3–8), medium risk (cumulative score 9–13), and high risk (cumulative score ≥14). For statistical analysis, cases were categorized into low (including none/low risk), medium, and high BEWE risk levels. Prior to clinical data collection, examiner calibration for the BEWE assessment was performed on a sample of 17 patients (∼10% of the study cohort) who were re-examined after a 2-week interval to minimize memory bias, yielding a Cohen’s kappa coefficient of κ=0.85, which confirmed excellent intra-examiner agreement. The clinical examination was performed by the same operator, the highest BEWE score per sextant was recorded, and the cumulative score was classified into three risk categories for dental erosion development as follows: low, medium, and high.

Before the clinical examination, patients were instructed to refrain from eating, drinking, smoking, tooth brushing, and using mouthwash for at least one hour prior to the appointment. Unstimulated saliva was collected by expectoration into a single-use cup and analyzed immediately chairside. No external transport or prolonged storage was required. Two salivary parameters were assessed: salivary pH and salivary buffering capacity.

Salivary pH was measured by immersing a pH strip in the saliva sample for 10 s, with colorimetric reading against a reference chart. Results were interpreted as: very acidic (pH 5.0–5.8, red/orange), moderately acidic (pH 6.0–6.6, yellow), or healthy (pH 6.8–7.8, green). Salivary buffering capacity was evaluated using the Saliva Check BUFFER single-use test kit (GC Corporation, Japan; European distribution: GC Europe). A drop of saliva was applied to each of the three reactive zones of the test strip. The strip was immediately rotated to 90° to eliminate excess saliva, and the result was read after 2 minutes. Each zone was scored by color (grey = 4 pts; grey/blue = 3 pts; blue = 2 pts; red/blue = 1 pt; red = 0 pts), yielding a total score of 0–12, interpreted as: very low buffering capacity (0–5), low (6–9), or normal/elevated (10–12).

All collected data were entered into a database, coded, verified for consistency, and anonymized prior to analysis. The data obtained was statistically analyzed with the help of SPSS-Statistical Package for the Social Sciences version 29.0 for Windows. Associations between BEWE risk categories and categorical variables (age group, systemic conditions, dietary habits, and salivary parameters) were assessed using the chi-square test (χ^2^). The strength of associations was quantified using odds ratios (OR) with 95% confidence intervals. The primary outcome variable, BEWE risk level, was treated as an ordered categorical variable with three levels: low risk (cumulative score ≤8, reference level), medium risk (cumulative score 9–13), and high risk (cumulative score ≥14). Multivariable analysis was conducted using cumulative ordinal logistic regression. The proportional odds assumption was evaluated using the Test of Parallel Lines, confirming that the slope coefficients were consistent across cumulative logit thresholds (p=0.896). Parameter estimates (B), p-values, and Odds Ratios (OR) with 95% confidence intervals were calculated to evaluate the cumulative likelihood of transitioning to a higher BEWE risk tier Statistical significance was set at *p* < 0.05.

Using G*Power 3.1.9.7 software (Heinrich-Heine-Universität Düsseldorf, Düsseldorf, Germany), an a priori power analysis was conducted to determine the optimal sample size for the primary comparisons. For a two-tailed $\chi^{2}$ test of independence (or logistic regression), setting a significance level of α=0.05, a desired power of 1−β=0.80, and assuming a medium effect size (w=0.30/OR=2.0, based on previous epidemiological studies on BEWE categories), the minimum required sample size was determined to be N=134 participants. Our enrolled cohort of n=170 exceeded this minimum, ensuring sufficient power for univariate bivariate analyses.

## 3. Results

The current study included 170 patients, 92 females (54.12%) and 78 males (45.88%). The risk of dental erosion (BEWE) as well as the salivary buffering capacity were assessed.

**Figure 1:**
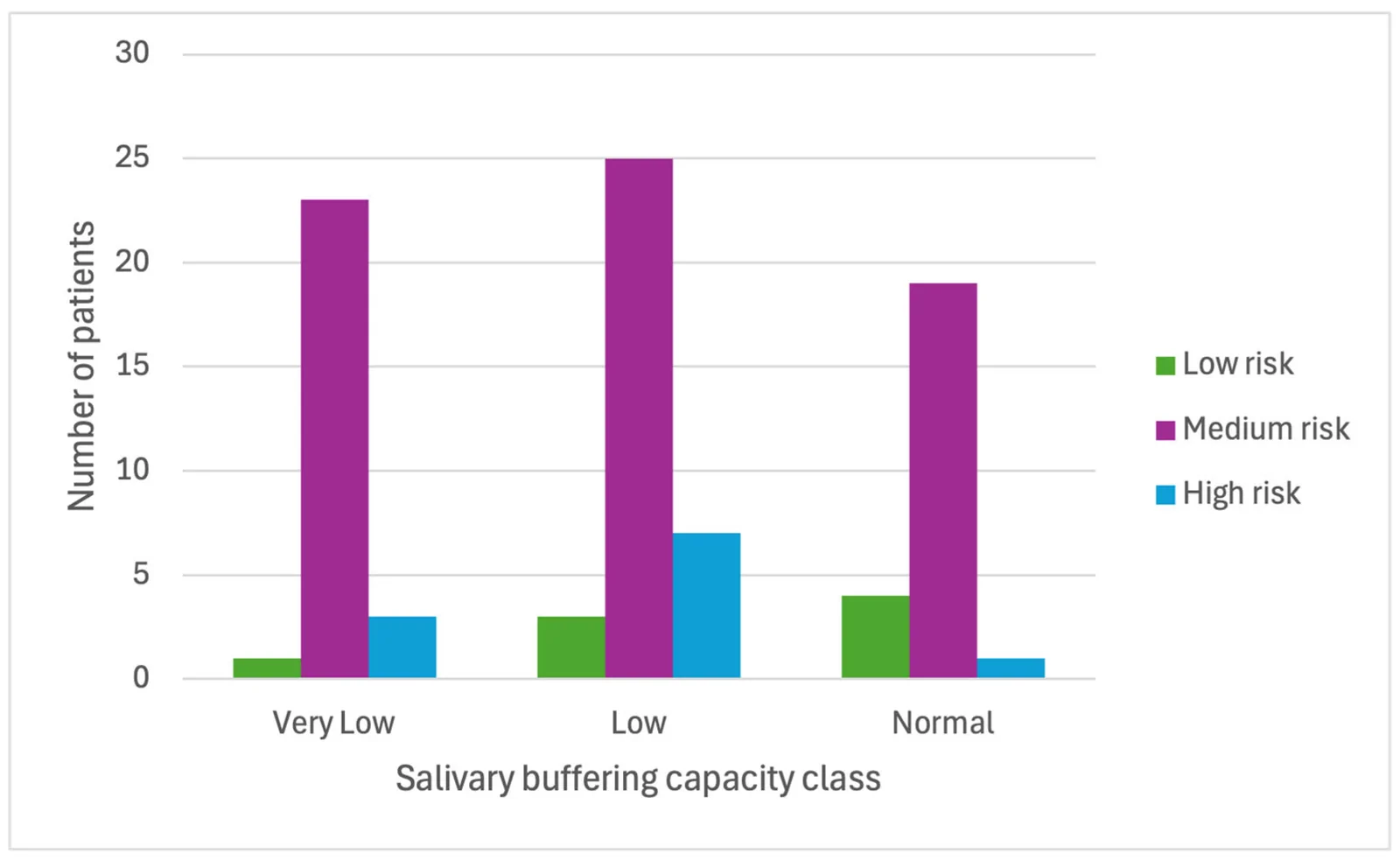
Relationship between salivary buffering capacity and the risk of dental erosion (BEWE).

As illustrated in Figure 1, most patients were categorized within the low BEWE risk group. Patients with low salivary buffering capacity showed a higher proportion of cases classified as having an increased risk of dental erosion. In contrast, higher buffering capacity was associated with lower BEWE risk categories, suggesting a potential protective effect.

The study population was stratified into 3 age categories. A moderate, statistically significant positive monotonic correlation between age and BEWE risk levels was identified (Table 1), indicating that higher age categories are directly associated with increased cumulative dental erosion risk (Chi-Square Test: $\chi^{2}=25.84,\;df=4,\;p<0.001$, Spearman’s Rank Correlation: ρ=0.382, p<0.001).

The association between BEWE risk and the patients’ general health status was analyzed, as presented in Table 2. A statistically significant relationship was identified only for gastroesophageal reflux disease (GERD) and eating disorders (*p* < 0.05), indicating that BEWE risk was significantly associated with the presence of these conditions. Furthermore, patients diagnosed with GERD or eating disorders were more frequently classified within the medium BEWE risk category.

Dietary factors were evaluated in relation to BEWE risk, as summarized in Table 3. Among the factors analyzed, only the consumption of carbonated beverages showed a statistically significant association with BEWE risk (*p* < 0.05). Patients with higher intake of carbonated drinks were more frequently classified in the higher BEWE risk categories, indicating a positive relationship between consumption and dental erosion severity.

Ordinal logistic regression analysis identified age, gastroesophageal reflux disease (GERD), difficulty swallowing, and selected medication use as the main factors associated with BEWE risk. Increasing age was associated with a higher likelihood of belonging to a more severe BEWE category, while the absence of GERD and of difficulty swallowing was associated with lower risk. Male sex showed a tendency toward higher BEWE risk, although this association did not reach statistical significance. Medication-related variables, particularly anti-asthma medication and aspirin use, were also associated with BEWE risk. Detailed regression coefficients, odds ratios, confidence intervals, and *p*-values are presented in Table 4.

Among symptom-related variables, difficulty swallowing and digestive burns were the most relevant contributors to BEWE risk, whereas several other clinical and lifestyle factors were not significantly associated with the outcome. Overall, the regression model supported the role of age and reflux-related symptomatology as the strongest predictors of increased erosive wear risk in this cohort.

Medication use also appeared to influence BEWE risk, particularly for anti-asthma medication and aspirin, whereas other evaluated variables did not show statistically significant associations in the adjusted model.

Other variables, including asthma, autoimmune diseases, eating disorders, and lifestyle factors such as pool exposure and oral irrigator use, did not demonstrate statistically significant associations with BEWE risk in the multivariable analysis. The parallel lines test indicated that the proportional odds assumption was satisfied, supporting the adequacy of the ordinal model.

**Figure 2:**
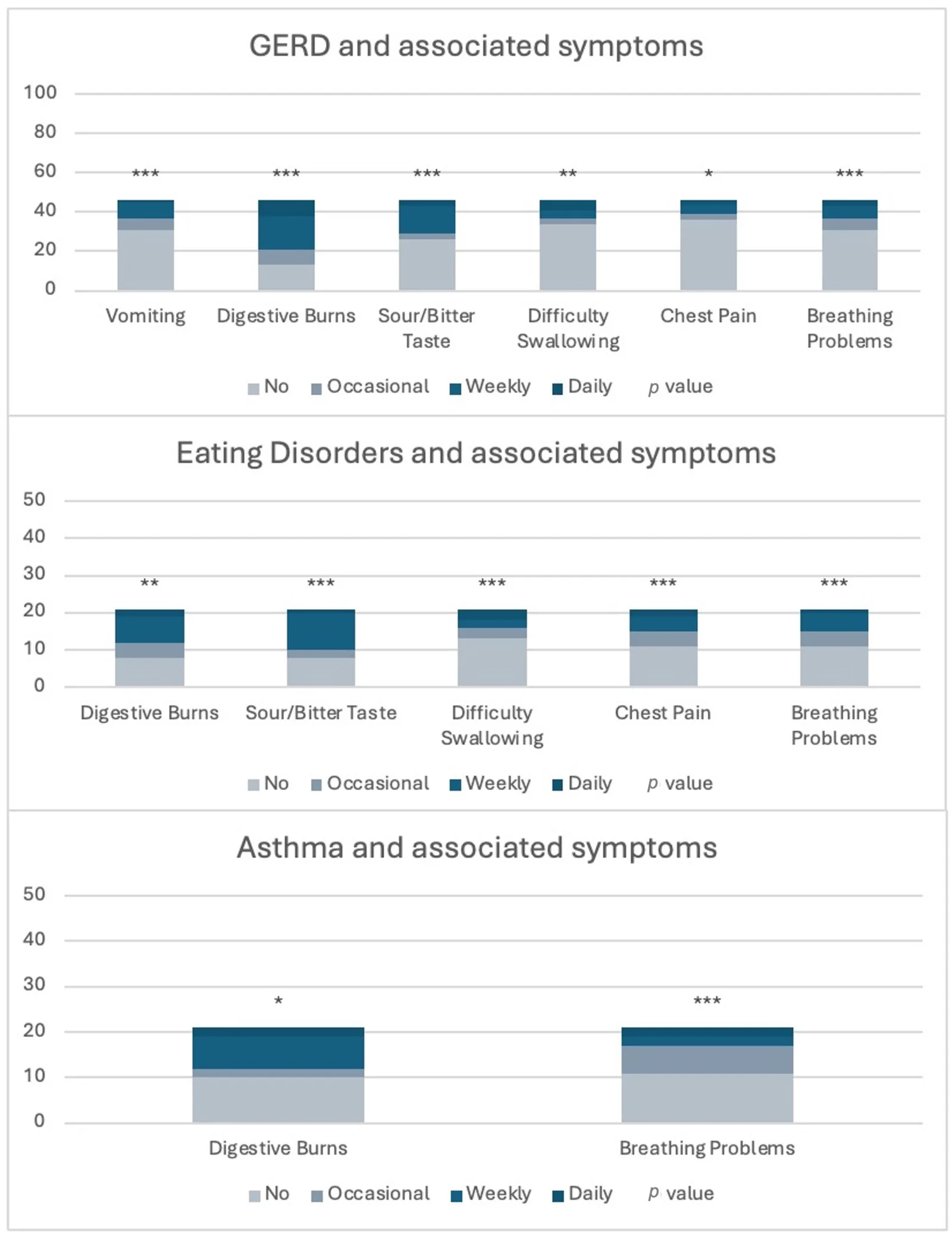
Stacked bar charts showing the distribution of reported symptom frequency categories (No, Occasional, Weekly, Daily) for significant associations between systemic conditions and symptoms. Statistical significance is indicated by asterisks (* *p* < 0.05; ** *p* < 0.01; *** *p* < 0.001).

Analysis of the association between systemic conditions and gastrointestinal/respiratory symptoms revealed several statistically significant relationships (*p* < 0.05) as shown in Figure 2. A strong association was identified between gastroesophageal reflux disease (GERD) and multiple symptoms, including vomiting, digestive burns, sour/bitter taste, difficulty swallowing, chest pain, and breathing problems. GERD demonstrated the highest number of statistically significant associations across all evaluated symptoms, suggesting it may be a primary contributing factor in the pathophysiology of dental erosion-related manifestations. This consistent pattern highlights the central role of GERD in both gastrointestinal and extra-esophageal symptom presentation.

Similarly, eating disorders showed statistically significant associations with digestive burns, sour/bitter taste, difficulty swallowing, chest pain, and breathing problems. These findings suggest that disordered eating behaviors may contribute substantially to symptom profiles associated with dental erosion, particularly through repeated exposure to intrinsic acids. In addition, asthma was significantly associated with digestive burns and breathing problems, highlighting a possible overlap between respiratory pathology and gastrointestinal symptomatology.

No statistically significant associations were observed between autoimmune diseases or alcoholism and the investigated symptoms (*p* > 0.05). However, this lack of significance may be influenced by the relatively small number of patients in these subgroups, which could limit statistical power.

## 4. Discussion

The present cross-sectional study highlights a multifactorial etiology for erosive tooth wear. While bivariate analyses identified associations with age, GERD, eating disorders, and carbonated beverage intake, multivariable adjustment confirmed that increasing age, GERD, and reflux-related symptoms represent the strongest independent predictors in this cohort. Variables such as eating disorders and carbonated beverage consumption lost statistical significance after multivariable adjustment, underscoring the complex confounding among systemic and behavioral factors.

The overall distribution of BEWE risk in our cohort reflects a predominantly low-risk population, a pattern that has also been reported in studies conducted in general or early detection clinical settings. A 2025 cross-sectional study using the BEWE index among dental students found that participants were predominantly classified within the no-risk or low-risk categories, with no individuals presenting advanced erosive wear, suggesting that low-severity erosive changes are common in populations without major systemic or behavioral risk factors [11]. The predominance of low-risk BEWE scores in our sample may reflect the inclusion of a broad, relatively unselected patient population attending a prosthodontic clinic, where early-stage erosive lesions are more likely to be identified before substantial hard tissue loss develops. Similarly, a 2024 cross-sectional study among Colombian adolescents reported that 84.3% of participants presented BEWE scores corresponding to low-risk categories, reinforcing that mild erosive wear is the most frequently observed presentation when tooth wear is assessed systematically in diverse populations [12].

One of the most robust findings of this study is the association between age and BEWE risk. The proportion of patients in the high-risk category increased progressively with advancing age, illustrating a clear cumulative exposure pattern. A narrative review of clinical studies addressing tooth wear in older adults highlighted the progressive accumulation of erosive lesions with age, identifying medical conditions, hyposalivation, and dietary habits as major contributing factors, while emphasizing the importance of early diagnosis to avoid complex restorative procedures [13]. A cross-sectional clinical study using the BEWE index confirmed that tooth wear increases significantly with advancing age group (*p* < 0.01), with the proportion of patients classified in higher risk levels rising consistently from young adults to older age categories [14]. These findings reinforce the importance of proactive erosion monitoring in older patient cohorts, particularly those presenting with concurrent systemic risk factors.

Male sex was associated with higher BEWE risk in our sample. This finding is supported by a 10-year prospective longitudinal study conducted in Romanian young adults, which found that males exhibited a higher incidence of dental erosion compared to females (29.9% vs. 17.2%; RR = 1.74, *p* < 0.001), with greater enamel loss observed on palatal surfaces [15]. Several behavioral and lifestyle mechanisms may underline this disparity. A 2022 study comparing oral health behaviors between men and women found that males were less likely to attend preventive dental visits and more frequently sought dental care only for urgent treatment needs, suggesting systematic differences in preventive oral health engagement between sexes [16]. Furthermore, eating behaviors demonstrate both biological sex differences and gender-related influences associated with cultural norms, education, and occupational factors, with men generally consuming more acidic foods and beverages that may increase the risk of dental erosion [17]. These combined behavioral and dietary patterns, potentially amplified by occupational acid exposure, may collectively explain the elevated erosion risk observed in male patients in our cohort.

The strongest systemic association identified in this study was between GERD and BEWE risk, confirming GERD as a major intrinsic risk factor for erosive tooth wear. Recent meta-analytic evidence has confirmed GERD as one of the strongest systemic predictors of erosive tooth wear, with affected patients demonstrating substantially higher prevalence rates of dental erosion than healthy controls [18]. Repeated exposure of dental tissues to gastric acid (pH 1–2) promotes irreversible demineralization that exceeds the protective buffering capacity of saliva. A 2025 systematic review further confirmed a significantly higher prevalence of dental erosion in adult populations with GERD or laryngopharyngeal reflux and emphasized the importance of early identification and management of underlying reflux conditions [19].

Additionally, a clinical study using the BEWE index demonstrated that the extent of dental erosion was positively associated with GERD symptom severity (B = 0.585; 95% CI: 0.21–0.96) and suggested that dental erosion may occasionally represent the only clinical manifestation of undertreated GERD [20]. Taken together, these findings strongly support the integration of GERD screening into routine dental consultations, particularly when characteristic erosive patterns such as palatal surface involvement are identified.

Eating disorders were associated with BEWE risk in this study and showed associations with a broad range of gastrointestinal and respiratory symptoms, highlighting the systemic complexity of these conditions and their oral manifestations. These findings are consistent with recent systematic reviews. Nijakowski et al. (2023) [6] and Valeriani et al. (2024) [7] documented elevated rates of erosive lesions in patients with bulimia nervosa and anorexia nervosa, with the strongest associations observed in bulimic patients due to repeated self-induced vomiting and chronic acid exposure of dental surfaces. Oral manifestations may represent the first clinical indicators of a disordered eating pattern, highlighting the important role of dental practitioners in early detection and referral to multidisciplinary care.

Patients with low salivary buffering capacity were more frequently classified in higher BEWE risk categories, supporting the protective role of saliva against erosive tooth wear. A 2025 cross-sectional study among dental students confirmed that saliva plays an important role in modulating erosive wear through its buffering capacity and remineralization potential, with salivary flow rate, pH, and buffering capacity identified as key susceptibility factors influencing the progression of erosive wear [11]. The protective mechanisms of saliva against erosion are multifactorial. An experimental study published in 2022 demonstrated that saliva contributes to oral clearance, dilution, and buffering of acids after their introduction into the oral cavity, and that the presence of saliva significantly reduced enamel and dentine surface loss under both erosive and combined erosion-attrition conditions compared to saliva-free environments [21]. In addition, the acquired salivary pellicle acts as a protective diffusion barrier that limits direct acid contact with enamel surfaces. Although the association between buffering capacity and BEWE risk did not remain significant in the multivariate model, likely due to sample size limitations, the descriptive trend observed remains clinically relevant and warrants further investigation in larger prospective cohorts.

Among all dietary variables evaluated, only carbonated beverage consumption was associated with BEWE risk. A 2023 systematic review concluded that overconsumption of carbonated acidic beverages substantially increases the probability of dental erosion, resulting in structural disintegration and reduction in enamel mechanical properties, while noting that the pH of most commercially available carbonated drinks falls below the critical threshold for enamel demineralization [22]. The erosive impact is further modulated by factors not captured in a simple frequency questionnaire, including total volume consumed, duration of oral contact, sipping behavior, and beverage temperature. It is worth noting that the relationship between consumption frequency and BEWE risk in our data was not strictly monotonic: patients reporting occasional consumption showed a more favorable risk distribution compared to those reporting no consumption at all. This finding may reflect residual confounding, such as dietary substitution with other acidic beverages in the “no carbonated drinks” group and should therefore be interpreted cautiously.

The absence of statistically significant associations with citrus fruit/apple consumption and energy drinks should not be interpreted as evidence against their erosive potential. A 2025 systematic review on sports drinks and dental erosion emphasized that the erosive effect of acidic beverages is strongly influenced by consumption patterns such as sipping behavior, volume, and frequency, variables that simple categorical questionnaires often fail to capture adequately, potentially leading to underestimation of their effect in cross-sectional studies [8]. Additionally, the energy drink subgroup in our sample was highly skewed toward non-consumption, with only a very small number of patients reporting daily intake, thereby substantially limiting statistical power to detect an association. Protective behaviors such as rinsing with water or using a straw may further attenuate erosive effects in some individuals, contributing to the null findings observed.

Furthermore, several limitations must be acknowledged. The study was conducted at a single university clinic using consecutive sampling, which may limit the generalizability of the findings and introduce selection bias. Data regarding dietary habits and medical history were collected through self-report, introducing potential recall and social desirability bias, as well as possible misclassification of underdiagnosed or underreported conditions. Some disease subgroups were very small (alcoholism n = 2, autoimmune diseases *n* = 3), precluding meaningful statistical inference for these conditions and likely reflecting insufficient statistical power rather than a true absence of association; similarly, some exposure categories included very few participants. All clinical examinations were performed by a single examiner, which reduced inter-observer variability but did not allow assessment of inter-examiner reproducibility. The cross-sectional design precludes causal inference, and all reported relationships should therefore be interpreted as correlational. Additionally, a statistical limitation of this study lies in the multivariable regression model, which evaluated a large number of potential predictor variables relative to the sample size (n=170). This low ratio of sample size to predictors introduces a risk of model overfitting and potential instability in the parameter estimates. Consequently, the findings from the regression analysis should be interpreted with caution and considered hypothesis-generating rather than confirmatory. Future prospective studies with larger, multi-center cohorts are needed to confirm these predictors using more parsimonious variable selection strategies. Another methodological consideration in our multivariable regression model involves potential multicollinearity between gastroesophageal reflux disease (GERD) and its associated gastrointestinal symptoms (such as digestive burns, sour/bitter taste, and difficulty swallowing). As shown in Figure 2, these symptoms strongly cluster within GERD-diagnosed individuals, acting as direct clinical surrogates or mediators of the underlying condition. Concurrently including both GERD and individual reflux symptoms in the ordinal model introduced degree-of-freedom inflation and collinearity, which contributed to inflated standard errors and wide confidence intervals for certain symptom parameters (e.g., sour/bitter taste). While these symptoms were retained to evaluate their potential additive value in un-diagnosed patient screenings, their specific regression coefficients should be interpreted cautiously due to collinearity with GERD. Furthermore, while our questionnaire assessed general respiratory symptoms and asthma, the study did not specifically screen for sleep-disordered breathing (SDB) or obstructive sleep apnea (OSA). SDB is an established potential trigger for nocturnal gastroesophageal reflux, which can in turn exacerbate intraoral acid exposure and erosive tooth wear. The absence of validated SDB/OSA screening protocols represents an unmeasured confounding factor in our symptom analysis. Future interdisciplinary studies investigating dental erosion should incorporate SDB evaluations to clarify the potential pathway connecting sleep-disordered breathing, nocturnal reflux, and enamel demineralization. Additionally, Body Mass Index (BMI) and degree of obesity were not recorded in this patient cohort. Given that elevated BMI is a recognized risk factor for gastroesophageal reflux disease (GERD) and intra-abdominal pressure increases, the lack of BMI measurements represents an unmeasured confounding factor in our regression models. Another limitation is represented by the lack of investigation of smoking habits; therefore, future studies should include detailed smoking-related variables (e.g., smoking status, frequency, duration, and pack-years) to better evaluate their independent effects on salivary characteristics and dental erosion risk. Furthermore, future investigations could consider stratified analyses or adjustment for smoking exposure in multivariable models to reduce residual confounding and improve the interpretation of salivary test results. Finally, salivary assessment was performed using a point-of-care colorimetric kit which, although standardized and clinically practical, may provide lower precision than laboratory-based salivary analysis, and salivary parameters were measured at a single time point despite their potential variability according to hydration, circadian factors, and recent intake.

## 5. Conclusions

Dental erosion is a clinically relevant multifactorial condition shaped by the combined influence of age, systemic health, dietary exposure, and salivary protective factors. While bivariate analysis linked diet and eating disorders, only age, GERD, and symptoms independently predicted erosion. While the overall regression model highlighted age and reflux-related symptoms as potential major predictors, these associations require validation in larger populations before definitive predictive conclusions can be established.

## Figures and Tables

**Table 1:** Association between age categories and BEWE risk levels ($\chi^{2}$ test).

Age	Risk BEWE			Total	*p*-Value
	Low *n* (%)	Medium *n* (%)	High *n* (%)		
Young adults (<30 yo)	29 (17.1)	34 (20)	2 (1.2)	65 (38.24)	0
Middle aged (30–50 yo)	6 (3.5)	39 (22.9)	4 (2.4)	49 (28.82)	
Older adults	9 (5.3)	34 (20)	13 (17.6)	56 (32.94)	
Total	44 (22.88)	107 (62.94)	19 (11.18)		

**Table 2:** Association between systemic health conditions and BEWE risk levels ($\chi^{2}$ test).

Disease	Risk BEWE			Total	*p* Value
	Low *n* (%)	Medium *n* (%)	High *n* (%)		
Asthma	5 (23.81)	13 (61.90)	3 (14.29)	21 (12.35)	0.88
Autoimmune Diseases	0 (0)	2 (66.67)	1 (33.33)	3 (1.76)	0.34
Alcoholism	0 (0)	2 (100)	0 (0)	2 (1.18)	0.55
Gastroesophageal Reflux Disease	3 (6.52)	29 (63.04)	14 (30.43)	46 (27.06)	0
Eating Disorders	3 (14.29)	12 (57.14)	6 (28.57)	21 (12.35)	0.02

**Table 3:** Association between dietary factors and BEWE risk levels ($\chi^{2}$ test).

Diet		Risk BEWE			Total	*p* Value
		Low *n* (%)	Medium *n* (%)	High *n* (%)		
Carbonated Drinks	No	14 (8.20)	59 (34.70)	10 (5.90)	83 (48.82)	0.004
	Occasional	11 (6.50)	6 (3.50)	2 (1.20)	19 (11.18)	
	Weekly	15 (8.80)	22 (12.90)	3 (1.80)	40 (23.53)	
	Daily	4 (2.40)	20 (11.80)	4 (2.40)	28 (16.47)	
Citrus/Apples	No	9 (5.30)	21 (12.40)	5 (2.90)	35 (20.59)	0.377
	Occasional	6 (3.50)	7 (4.10)	3 (1.80)	16 (19.41)	
	Weekly	13 (7.60)	37 (21.80)	2 (1.20)	52 (30.59)	
	Daily	16 (9.40)	42 (24.70)	9 (5.30)	67 (39.41)	
Energy Drinks	No	35 (20.60)	96 (56.50)	19 (11.20)	150 (88.24)	0.141
	Occasional	6 (3.50)	4 (2.40)	0 (0)	10 (5.88)	
	Weekly	3 (1.80)	5 (2.90)	0 (0)	8 (4.71)	
	Daily	0 (0)	2 (1.20)	0 (0)	2 (1.18)	

**Table 4:** Ordinal logistic regression analysis of factors associated with BEWE risk.

				*p*	Odds Ratio
Model Fitting Information				<0.001	
Goodness-of-Fit				0.06	
Pseudo R-Square		McFadden	0.333		
Parameter Estimates		Age	0.051	<0.001	1.052323
		Sex = Male	0.776	0.058	2.172764
		Sex = Female	0 a		
		Asthma = No	1.721	0.095	5.590116
		Asthma = Yes	0 a		
		Autoimmune Diseases = No	0.119	0.946	1.12637
		Autoimmune Diseases = Yes	0 a		
		Alcoholism = No	−1.432	0.525	0.238831
		Alcoholism = Yes	0 a		
		GERD = No	−1.721	0.003	0.178887171
		GERD = Yes	0 a		
		Eating Disorders = No	−0.477	0.519	0.620643
		Eating Disorders = Yes	0 a		
		Vomiting = No	0.425	0.897	1.52959
		Vomiting = Occasional	−0.363	0.914	0.695586
		Vomiting = Weekly	1.677	0.624	5.349483
		Vomiting = Daily	0 a		
		Digestive Burns = No	−2.953	0.015	0.052183
		Digestive Burns = Occasional	−2.484	0.067	0.083409
		Digestive Burns = Weekly	−2.426	0.074	0.08839
		Digestive Burns = Daily	0 a		
		Sour/Bitter Taste = No	3.033	0.233	20.75942
		Sour/Bitter Taste = Occasional	3.454	0.193	31.62665
		Sour/Bitter Taste =Weekly	4.289	0.09	72.89354
		Sour/Bitter Taste = Daily	0 a		
		Difficulty Swallowing = No	−3.31	0.013	0.036516
		Difficulty Swallowing = Occasional	−4.523	0.006	0.010856
		Difficulty Swallowing = Weekly	1.199	0.478	3.316798
		Difficulty Swallowing = Daily	0 a		
		Chest Pains = No	0.496	0.919	1.64214
		Chest Pains = Occasional	−0.545	0.912	0.579842
		Chest Pains = Weekly	−2.762	0.575	0.063165
		Chest Pains = Daily	0 a		
		Breathing Problems = No	1.326	0.561	3.765949
		Breathing Problems = Occasional	2.032	0.4	7.62933
		Breathing Problems = Weekly	1.435	0.584	4.199645
		Breathing Problems = Daily	0 a		
		Antiacids = No	−0.31	0.686	0.733447
		Antiacids = Yes	0 a		
		Antiasthma = No	−2.385	0.035	0.092089
		Antiasthma = Yes	0 a		
		Aspirin = No	−1.446	0.032	0.23551
		Aspirin = Yes	0 a		
		Bruxism = No	−0.012	0.982	0.988072
		Bruxism = Yes	0 a		
		Muscle Fatigue = No	−0.498	0.524	0.607745
		Muscle Fatigue = Yes	0 a		
		Whitening Paste = No	−0.802	0.125	0.448431
		Whitening Paste = Yes	0 a		
		Oral Irrigator = No	0.391	0.351	1.478459
		Oral Irrigator = Yes	0 a		
		Pool = No	−0.726	0.23	0.48384
		Pool = Yes	0 a		
		Work Environment = No	0.749	0.321	2.114884
		Work Environment = Yes	0 a		
		Salivary pH (5–5.8)	5.820	<0.001	337.31
		Salivary pH (6–6.8)	3.650	<0.001	38.47
		Salivary pH (≥7)	0 a	1.00	1.00
		Buffering Capacity = Low	0.845	0.182	2.33
		Buffering Capacity = Medium	0.412	0.451	1.51
		Buffering Capacity = High	0 a	1.00	
Test of Parallel lines				0.896	

a. This parameter is set to zero because it is the reference category.

## Data Availability

Dataset available on request from the authors.
